# Web-Based Tool (FH Family Share) to Increase Uptake of Cascade Testing for Familial Hypercholesterolemia: Development and Evaluation

**DOI:** 10.2196/32568

**Published:** 2022-02-15

**Authors:** Hana Bangash, Ahmed Makkawy, Justin H Gundelach, Alexandra A Miller, Kimberly A Jacobson, Iftikhar J Kullo

**Affiliations:** 1 Mayo Clinic Rochester, MN United States; 2 Saharafox Creative Agency Rochester, MN United States

**Keywords:** familial hypercholesterolemia, cascade testing, communication, genetic counselors, digital tools, website, usability, user experience, public health

## Abstract

**Background:**

Familial hypercholesterolemia, a prevalent genetic disorder, remains significantly underdiagnosed in the United States. Cascade testing, wherein individuals diagnosed with familial hypercholesterolemia— probands—contact their family members to inform them of their risk for familial hypercholesterolemia, has low uptake in the United States. Digital tools are needed to facilitate communication between familial hypercholesterolemia probands and their family members and to promote sharing of familial hypercholesterolemia–related risk information.

**Objective:**

We aimed to create and evaluate a web-based tool designed to enhance familial communication and promote cascade testing for familial hypercholesterolemia.

**Methods:**

A hybrid type 1 implementation science framework and a user-centered design process were used to develop an interactive web-based tool—FH Family Share—that enables familial hypercholesterolemia probands to communicate information about their familial hypercholesterolemia diagnosis with at-risk relatives. Probands can also use the tool to draw a family pedigree and learn more about familial hypercholesterolemia through education modules and curated knowledge resources. Usability guidelines and standards were taken into account during the design and development of the tool. The initial prototype underwent a cognitive walkthrough, which was followed by usability testing with key stakeholders including genetic counselors and patients with familial hypercholesterolemia. Participants navigated the prototype using the think-aloud technique, and their feedback was used to refine features of the tool.

**Results:**

Key themes that emerged from the cognitive walkthrough were design, format, navigation, terminology, instructions, and learnability. Expert feedback from the cognitive walkthrough resulted in a rebuild of the web-based tool to align it with institutional standards. Usability testing with genetic counselors and patients with familial hypercholesterolemia provided insights on user experience, satisfaction and interface design and highlighted specific modifications that were made to refine the features of FH Family Share. Genetic counselors and patients with familial hypercholesterolemia suggested inclusion of the following features in the web-based tool: (1) a letter-to-family-member email template, (2) education modules, and (3) knowledge resources. Surveys revealed that 6 of 9 (67%) genetic counselors found information within FH Family Share very easy to find, and 5 of 9 (56%) genetic counselors found information very easy to understand; 5 of 9 (56%) patients found information very easy to find within the website, and 7 of 9 (78%) patients found information very easy to understand. All genetic counselors and patients indicated that FH Family Share was a resource worth returning to.

**Conclusions:**

FH Family Share facilitates communication between probands and their relatives. Once informed, at-risk family members have the option to seek testing and treatment for familial hypercholesterolemia.

## Introduction

Familial hypercholesterolemia is one of the most common genetic disorders worldwide and is a significant public health burden [1]. With a prevalence of approximately 1 in 250, it is estimated that, in the United States, there are 1.3 million individuals with familial hypercholesterolemia and only 10% have been diagnosed [2,3]. Familial hypercholesterolemia is a treatable disorder, yet due to the lack of awareness, patients remain at significantly increased risk of premature coronary heart disease due to elevated low-density lipoprotein cholesterol levels starting early in life [4-6]. Therefore, increasing familial hypercholesterolemia detection by using cascade testing is important to prevent coronary heart disease and reduce familial hypercholesterolemia–related morbidity and mortality.

Cascade testing, wherein individuals diagnosed with familial hypercholesterolemia (probands) contact their family members and encourage them to get tested for familial hypercholesterolemia, is the most cost-effective method of detecting new cases of familial hypercholesterolemia [7]. Cascade testing has been successfully implemented in a number of countries, most prominently in the Dutch health care system [8]. However, the uptake of cascade testing in the United States is low due to a number of barriers, such as the lack of a centralized and coordinated cascade testing program for familial hypercholesterolemia, the inability of health care providers to directly contact family members due to the Health Insurance Portability and Accountability Act Privacy Rule [9], complex family dynamics, and the burden placed on probands and health care providers in locating and contacting all at-risk family members [10]. In a recent study [11], only 28 of 240 (12%) familial hypercholesterolemia probands were able to enroll a family member for cascade testing, which highlights the low uptake of cascade testing in the United States.

Innovative digital tools have a central role to play in the implementation of genomic medicine by facilitating patient-centered care and decreasing disparities in health care by allowing increased access to care in diverse and underserved communities [12-14]. Well-designed digital tools may also be used to enhance the patient experience by encouraging patient engagement, promoting informed health care–related decisions, and increasing knowledge dissemination [13-15].

To develop digital tools for genomic medicine, it is necessary to obtain patient and provider input, ideally within an implementation science framework, to assess the tool’s potential effectiveness as well as institutional and individual level readiness for tool implementation. Patient and provider insights can be used to guide iterative refinements to digital tools and ensure smooth integration into clinical workflows. Hybrid study designs enable elements from both clinical effectiveness research and implementation science research to be blended to serve as a useful framework within which to gather stakeholder feedback and facilitate the translation of digital tools into practice [16].

## Methods

### Ethics

This study met institutional criteria for Quality Improvement and thus was not subject to review by the Institutional Review Board. The study was conducted from January 2018 to March 2021.

### Description of FH Family Share

We used a hybrid type 1 implementation science framework and a collaborative user-centered design process to develop an interactive website intended to facilitate communication between familial hypercholesterolemia probands and their family members. The design was guided by US Department of Health and Human Services Guidelines [17] and International Organization for Standardization Quality Standards for Usability [18]—guidelines that address web design and evaluation as well as user experience optimization for knowledge dissemination. Probands can share their familial hypercholesterolemia diagnosis with relatives via the letter-to-family-member email template. Email allows for faster communication than that using traditional postal mail; the email contains information on the relative likelihood of family members also having familial hypercholesterolemia (given that it is passed on as an autosomal dominant trait) and a recommendation to get tested. The proband can include information about the specific pathogenic variant that was found in their genetic test report, to facilitate family member genetic testing. The website also enables probands to build a family tree utilizing a pedigree tool (AboutMe, Mayo Clinic) to include first-degree relatives and document family members who may be at increased risk for familial hypercholesterolemia. Once a pedigree has been built, it can be accessed each time the user logs on. The web-based tool also has a section, called Learn, with educational modules on familial hypercholesterolemia–related topics: (1) What is Familial Hypercholesterolemia, (2) Familial Hypercholesterolemia Considerations in Children, (3) Genetics, (4) Genetic Testing Frequently Asked Questions, (5) Treatment, and (6) Additional Resources. Through these modules, users (probands or family members) can expand their knowledge on familial hypercholesterolemia and access informative patient education materials and links to other educational tools and websites.

In its current form, FH Family Share will be available on the Mayo Clinic intranet and the internet; all aspects of the web-based tool will be available for public access, with the exception of the pedigree tool which requires the use of a Mayo Clinic patient username and password, to ensure security of protected health information.

### Cognitive Walkthrough

The initial proof-of-concept prototype of the web-based tool was built by an external vendor using open-source PHP framework (Yii, version 2.0; Take The Wind). The graphical user interface was designed in HTML5 and CSS3 in conjunction with Jquery, connected to the MySQL database. It was developed as a responsive design for multiple screen sizes. The initial prototype underwent cognitive walkthrough—a technique applied to evaluate the usability of an app or system in the early stages of design. The cognitive walkthrough was conducted by 3 usability experts to assess exploratory learning—how well an end user can navigate the app for the first time without prior training [19-21]. The usability experts conducted the cognitive walkthrough at the Mayo Clinic Usability Laboratory and participated in 11 tasks that a first-time user was likely to undertake (Multimedia Appendix 1). The session lasted 2 hours and was observed by 5 study team members. Observers gave feedback on any difficulties they noted the experts encounter while navigating the website. The experts and observers discussed the feedback after each task, and points were documented on how to further improve the tool (Multimedia Appendix 2).

### Pilot Testing Program With Genetic Counselors

We evaluated user experience, satisfaction, and interface design of FH Family Share with usability testing that was informed by quality standards.[18]. A 1-year pilot testing program was launched in 2 phases (Rochester campus, Mayo Clinic), with genetic counselors as key stakeholders of the web-based tool. Purposive sampling was applied to recruit genetic counselors with varying clinical backgrounds, to obtain information-rich relevant insights on user experience and interface design. Genetic counselors were invited to participate in the usability testing sessions by email. A target sample size of 5 to 8 participants was established based on prior usability studies [22-25], in which approximately 80% to 85% of usability-related concerns were identified from the first 8 participants. We conducted 9 usability testing sessions in phase 1, and we conducted 7 usability testing sessions in phase 2.

Each usability session was 1-hour long; sessions were audiorecorded using a handheld recorder and transcribed using transcription software (version 2018; Otter.ai). Sessions were conducted by a user experience expert (AM) from an external company, to reduce any institutional or workflow driven biases. Study team members (HB and JHG) were also present during each session to observe and take notes. Participating genetic counselors were asked to assess educational content and interface design, as well as to provide insights on how FH Family Share would be integrated with their usual clinical workflows.

We used a digital platform (version 2011; InVisionApp Inc) to build an interactive clickable prototype of FH Family Share and to conduct user experience testing. The website prototype and 2 case scenarios (Multimedia Appendix 3) were presented to each genetic counselor; each participant was asked to verbalize their thoughts while navigating the prototype [26].

To facilitate feedback, genetic counselors were asked open-ended questions: “How do you feel each of the elements [on the website] might help you in your role?” “What do you think you will find when you click into this page?” “How might you share this website with a patient?” “What are some strengths and weaknesses [of the website]?” “Clicking that print button, what would you imagine would print out?”

**Figure 1:**
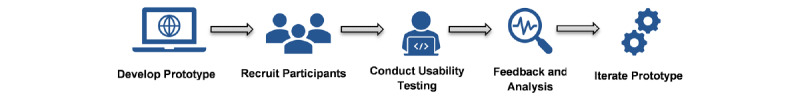
FH Family Share development and testing work flow.

Each round of user testing informed iterative refinements and modifications to the features of the prototype. User testing rounds were conducted until saturation was reached (ie, no new feedback was obtained) (Figure 1).

At the end of each usability testing session in phase 1, genetic counselors completed a 7-item satisfaction survey. Based on feedback from genetic counselors in phase 1, the survey was refined, and 4 additional questions were added (Multimedia Appendix 4); therefore, at the end of phase 2, genetic counselors completed an 11-item satisfaction survey.

### Evaluation With Patients

To obtain insights from end users, we conducted usability testing sessions over a 3-month period with patients with familial hypercholesterolemia. Patients were recruited using convenience sampling with a target sample size of 8 to 10 participants [22-25]. Patients with a confirmed pathogenic/likely pathogenic genetic variant for familial hypercholesterolemia and who previously participated in familial hypercholesterolemia–related research studies at Mayo Clinic were eligible for inclusion. Patients with a confirmed genetic diagnosis of familial hypercholesterolemia were selected to participate in this study as they had either gone through the process of cascade testing or were currently doing so and could therefore share important feedback and insights on the usefulness and relevance of FH Family Share in facilitating cascade testing. Patients received a request to participate in the study via 1 of 3 methods: the institutional patient portal, a telephone call, or an email. If patients indicated that they were interested in participating, they were contacted by phone to schedule the usability testing session.

Due to the COVID-19 pandemic, usability testing sessions were conducted using videoconferencing software (Zoom Video Communications Inc). Each session lasted 1 hour. Both audio and video from sessions were recorded; audiorecordings were transcribed using Office Word (version 2021; Microsoft Inc). Each session was conducted by a user experience expert (AM); study team members (HB, AA, and JHG) were also present during each session. Patients were emailed a link to access the clickable prototype of the web-based tool and were asked to think aloud when navigating the prototype. Patients did not receive any compensation for participating in the study.

At the start of each session, patients were prompted to describe their individual diagnostic journeys for familial hypercholesterolemia and provide insights on their family structure: “When were you diagnosed with FH (provide your age at the time of diagnosis or the calendar year)?” “Tell me about your experience receiving your FH diagnosis (mode and duration of communication, formats it was received in, and any patient education material you may have been given at the time).” “How did the way in which you received your diagnosis make you feel?” “What resources, if any, did you use to look up more information about FH?” “How did you find these resources (did you reach out to providers, search the internet etc)?” “How many first-degree relatives (parents, siblings, children) do you have?” “Did you share any of the information pertaining to your diagnosis with family members (if so, in what context or format)?” “If you did not share your diagnosis with family members, were there any barriers that prevented you from doing so?” “How many of your family members, that you know of, have completed testing for FH?” At the end of each session, patients were asked to complete an 11-item survey using web-based software (QualtricsXM) (Multimedia Appendix 5).

## Results

### Cognitive Walkthrough

Key themes that emerged from the cognitive walkthrough were design, format, navigation, terminology, instructions and learnability (Multimedia Appendix 6).

### Pilot Testing Program With Genetic Counselors

#### Usability Testing

Of 13 genetic counselors who were contacted, 9 consented to participate in phase 1. In phase 2, all 7 genetic counselors who were contacted agreed to participate (Multimedia Appendix 7).

**Figure 2:**
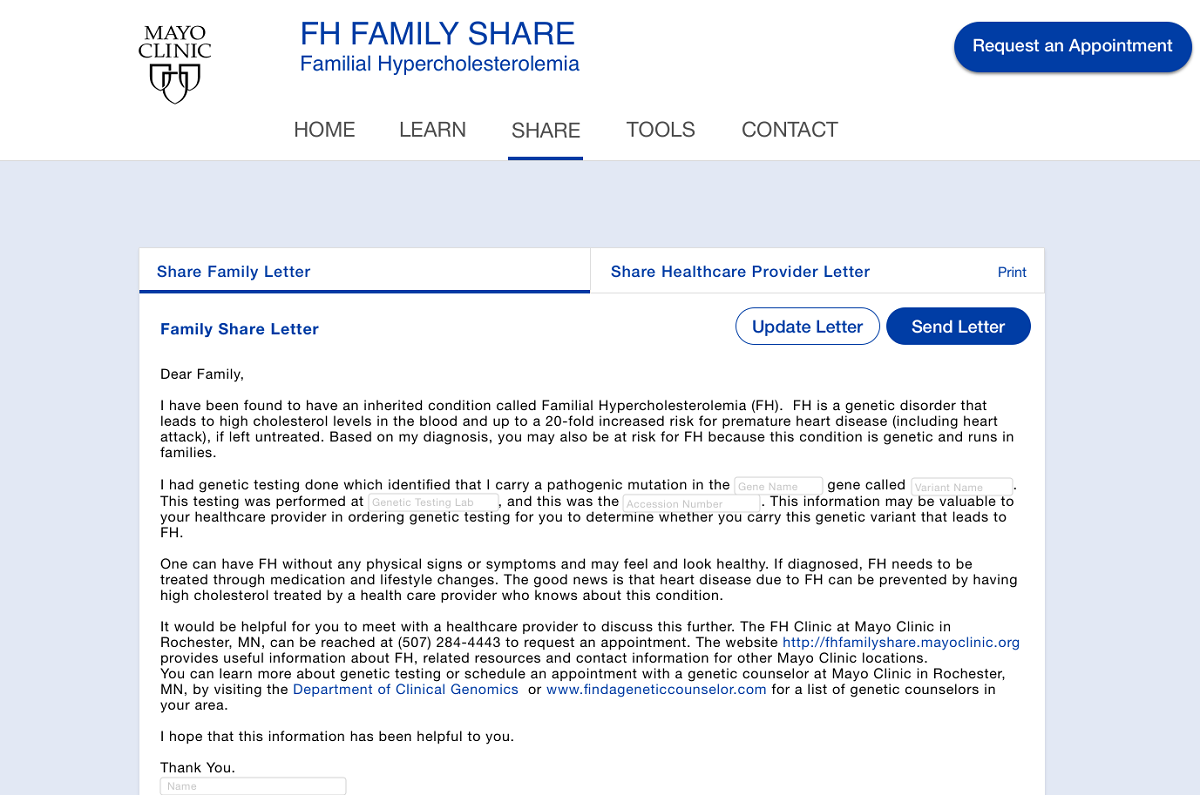
Screenshot of the template email on FH Family Share.

Usability testing with genetic counselors resulted in key workflow insights and highlighted specific modifications that could be made to FH Family Share. Genetic counselors found FH Family Share to be a welcome tool for use in disseminating knowledge and information to patients and facilitating communication on health risks information between patients and their family members. In current practice, once patients receives a diagnosis of familial hypercholesterolemia, they must use postal mail to send templated letters to family members informing them of their increased risk and recommending screening for familial hypercholesterolemia; genetic counselors indicated that the template email would aid patients in sharing their diagnosis and other pertinent information with family members and health care providers. In phase 1, 7 of 9 (78%) genetic counselors highlighted the need for a template email in which patients or genetic counselors could enter the name of the gene, pathogenic variant, and laboratory that conducted the genetic testing. Genetic counselors indicated that such information pertaining to the pathogenic variant would be valuable in enabling health care providers of family members determine which genetic testing was needed. Based on this feedback, the FH Family Share prototype was modified to include entry fields in the template email (Figure 2) that could be updated with relevant information on the pathogenic variant obtained from the patient’s genetic test report (this health information is not stored on the website).

**Figure 3:**
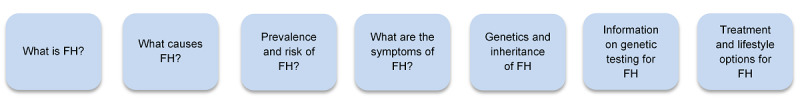
Sequence of educational topics.

Genetic counselors highlighted the importance of enabling patients to learn more about familial hypercholesterolemia because this would increase the likelihood of patients recommending cascade testing to their family members. However, all genetic counselors suggested rearranging the order of the content into a sequence that would be more in line with how they were likely to approach the conversation during their usual clinical workflows (Figure 3).

Additionally, 7 of 9 (78%) genetic counselors who participated in phase 1 of the pilot program recommended replacing stock photography and detailed medical images with patient-friendly material. This feedback led the study team to collaborate with graphic designers to develop medical illustrations for the physical manifestations of familial hypercholesterolemia, such as corneal arcus and tendon xanthomas [27].

Genetic counselors appreciated the integration of the pedigree tool with FH Family Share, particularly for its value in enabling patients to build their own pedigree and identify family members who may need to be screened for familial hypercholesterolemia, thereby facilitating earlier detection and treatment. Genetic counselors also valued the additional resources included on the website, such as links to patient education materials and videos.

#### Survey Results

The majority of the genetic counselors found information within the FH Family Share website very easy to find (phase 1: 6/9, 67%; phase 2: 6/7, 86%) and very easy to understand (phase 1: 5/9, 56%; phase 2: 6/7, 86%). In both phases of the pilot program (Table 1 and Table 2), all genetic counselors responded that the FH Family Share website was a resource worth returning to. In response to the questions added to the survey in phase 2, 5 of 7 (71%) genetic counselors agreed or completely agreed that the FH Family Share tool would ease their workflow during patient encounters, all genetic counselors agreed or completely agreed that the tool would likely improve follow-up patient care, 4 of 7 (57%) genetic counselors indicated that patients would likely use the Learn modules, and 2 of 7 (29%) genetic counselors indicated that patients were likely to send a letter to family members communicating their familial hypercholesterolemia diagnosis.

**Table 1:** Satisfaction survey responses from genetic counselors.

Item		Phase 1 (n=9), n (%)	Phase 2 (n=7), n (%)
**Overall, the information that you were asked to assess within the FH Family Share website was**			
	Very easy to find	6 (67)	6 (86)
	Somewhat easy to find	3 (33)	1 (14)
**Overall, the information that you found within the FH Family Share website was**			
	Very easy to understand	5 (56)	6 (86)
	Somewhat easy to understand	4 (44)	1 (14)
**Is the FH Family Share website a resource worth returning to?**			
	Yes	9 (100)	7 (100)
	No	0 (0)	0 (0)
**The FH Family Share website will ease my workflow in a patient encounter^a^**			
	Completely agree	—^b^	2 (29)
	Agree	—	3 (43)
	Neither agree nor disagree	—	2 (29)
**The FH Family Share website is likely to improve follow-up patient care^a^**			
	Completely agree	—	3 (43)
	Agree	—	4 (57)
	Neither agree nor disagree	—	0 (0)
**As a provider I feel the patient is most likely to^a,c^**			
	Use Learn modules as a knowledge resource	—	4 (57)
	Build a family tree using AboutMe	—	0 (0)
	Calculate risk of heart attack	—	1 (14)
	Send a letter to family members	—	2 (29)
	Use website information to discuss familial hypercholesterolemia with family members	—	0 (0)
**Do you find the website figures/images/diagrams useful?**			
	Yes	—	6 (86)
	No	—	0 (0)
	Other^d^	—	1 (14)

^a^The question was added for phase 2.

^b^No data for phase 1.

^c^Only 1 option could be selected.

^d^“Some were useful; more scientific diagrams were not as helpful from a patient perspective” [Participant 1, genetic counselor].

**Table 2:** Representative comments from genetic counselors in response to free-text questions. (Participant 4 did not provide free-text responses in phase 2.)

Item	Representative comments	
	Phase 1	Phase 2
What did you like most about the FH Family Share website?	“Solid basic information. Good patient reading level.” [Participant 4] “The education materials, getting contact info for the clinic.” [Participant 5] “The importance of informing family members and assistance to do this, especially the email.” [Participant 8]	“It was interactive and user-friendly. I think this will provide patients with a good tool to learn more about FH and how to easily share this information with their relatives.” [Participant 2] “Nice resource for families including the ability to share the resource and family letter within the website” [Participant 6]
What did you like least about the FH Family Share website?	“Ensure appropriate nomenclature is being used for heterozygous vs. homozygous FH.” [Participant 1] “Add more infographics about inheritance, genetics etc” [Participant 3] “The order of the modules under learn and the ambiguousness of ‘Discover’” [Participant 8]	“Images could use work” [Participant 1] “There is a lot of clicking into different tabs” [Participant 3]
What additional information or functionality would you like to see on the FH Family Share website?	“Personalization of email tool.” [Participant 2] “Next steps” for family members, how to find provider, how to share info with family.” [Participant 3]	“Use for other health care providers w/ limited experience in FH, incorporate Dutch Lipid Network, etc” [Participant 7]
What more could we do to improve the FH Family Share website?	“More meaningful Images.” [Participant 6] “A little more information about the About Me tool would be helpful prior to them starting it.” [Participant 7]	“Again, nothing further to add. Looks great!“ [Participant 2] “Make thing as concise as possible” [Participant 3] “Define terms or link to where they are defined.” [Participant 5]

### FH Family Share Evaluation With Patients

#### Usability Testing

Of 28 patients who were contacted, 13 responded with interest, and of those, 9 patients consented and participated in the usability testing sessions (Multimedia Appendix 8). The target sample size for participant recruitment was achieved. Patients who participated in the usability testing sessions had between 4 and 11 first-degree relatives; the majority of the patients (5 of 9, 56%) had shared their familial hypercholesterolemia diagnosis with all first-degree relatives, and additionally, 8 of the 9 patients had at least 1 or more first-degree relatives known to have undergone cholesterol testing or genetic testing.

Feedback obtained from patient usability testing sessions covered both content and interface design. Patients highlighted the need for a new section to be included in the *Learn* section of FH Family Share that would address the implications of familial hypercholesterolemia for children of variant-positive parents, the age at which children should be screened and treated, as well as the implications of having familial hypercholesterolemia when planning to start a family. Patients wanted to know the next best steps in these situations and emphasized the need to highlight the emotional motivation for getting tested for familial hypercholesterolemia and sharing a diagnosis with family members; children and grandchildren were identified as being the greatest motivators for patients. Additional feedback was obtained on the need to expand upon the information provided on proprotein convertase subtilisin/kexin type 9 inhibitors in the Treatment section by including details on how to obtain insurance approvals. Patients also shared input on interface design including need for a larger clearer font, to make content easier to read (using bullets instead of paragraphs), to use a less formal tone in the family letter, to add biographies to health care provider names on the Contact page, and to include more images and illustrations to make the website more interesting and engaging. Patient feedback led to further iterations of FH Family Share to incorporate their input.

#### Survey Results

Analysis of the satisfaction surveys revealed that 5 of 9 (56%) patients found information within the FH Family Share website very easy to find, and 7 of 9 (78%) found information very easy to understand (Table 3). When asked to share their perspective on how the website would impact patient care, 2 of 9 (22%) patients responded that it would significantly improve care, while 5 of 9 (56%) responded that it would somewhat improve care. The majority of patients agreed that the website would make it easier for them to understand and share their familial hypercholesterolemia diagnosis (completely agree: 5/9, 56%; agree: 4/9, 44%). When asked about the one activity they would most likely perform while using the website, 3 of 9 (33%) patients indicated that they would use the risk calculator to determine risk of heart attack. All patients agreed that the FH Family Share website was a resource worth returning to.

Patients also gave responses to 4 free-text survey questions (Table 4).

**Table 3:** Satisfaction survey responses from patients.

Survey questions			Patient responses, n (%)
**Overall, the information that you were asked to assess within the FH Family Share website was (n=9)**			
	Very easy to find	5 (56)	
	Somewhat easy to find	4 (44)	
**Overall, the information that you found within the FH Family Share website was (n=9)**			
	Very easy to understand	7 (78)	
	Somewhat easy to understand	2 (22)	
**How will the FH Family Share website impact patient care/follow-up care? (n=8)^a^**			
	Significantly improve	2 (22)	
	Somewhat improve	5 (56)	
	Neither improve nor worsen	1 (11)	
**The FH Family Share website will make it easier for patients like myself to understand and share an FH diagnosis (n=9)**			
	Completely Agree	5 (56)	
	Agree	4 (44)	
**As a patient, I would be most likely to (n=9)**			
	Use the Learn modules as a knowledge resource	2 (22)	
	Build a family tree using AboutMe	0 (0)	
	Calculate risk of a heart attack	3 (33)	
	Send a letter to family members	2 (22)	
	Use website information to discuss FH with family members	2 (22)	
**Do you find the FH Family Share website figures/images/diagrams useful? (n=9)**			
	Yes	8 (89)	
	No	1 (11)	
**Is the FH Family Share website a resource worth returning to? (n=9)**			
	Yes	9 (100)	
	No	0 (0)	

^a^Participant 6 did not provide a response to this survey question.

**Table 4:** Representative comments from patients with familial hypercholesterolemia in response to free-text questions.

Survey question	Representative comments
What did you like most about the FH Family Share website?	“The design intent is solid, and I did find more information regarding genetic variants than I had found doing research from other sources (Mayo, NIH, CDC)” [Participant 2] “It's nice to have all this information clearly compiled to be able to return and reference, especially for someone like me who has dealt with this diagnosis for most of my life but continue to learn more about it and how it will impact my health and potentially that of my family as I get older.” [Participant 5]
What did you like least about the FH Family Share website?	“There was no content that I disliked - the flow of the site could be improved a bit, as reviewed in the session.” [Participant 1] “Wish more of the links would have been active - but overall, the view and ease to move around was solid.” [Participant 2] “It was boring and not very visually stimulating.” [Participant 8]
What additional information or functionality would you like to see on the FH Family Share website?	“The risk stratification tool based on medications would be an awesome addition to this website.” [Participant 1] “As mentioned, perhaps more information geared toward younger patients, starting a family, thinking about getting children tested/when/why, and how risk can continue to be minimized over a lifetime.” [Participant 5] “Maybe interactive images.” [Participant 9]
What more could we do to improve the FH Family Share website?	“Create more emotional connection to why treatment is so important - perhaps by emphasizing risks. So many young people think they are invincible - as I did, as well - somehow make it more real, since you don't feel the effects of FH until it may be too late.” [Participant 2] “Pictures, diagrams, maybe address the holistic challenges folks with FH have in common.” [Participant 7]

## Discussion

### Principal Findings

Given the ubiquitous use of electronic health records, the internet, and smartphones, digital tools can serve a central role in the delivery and implementation of genomic medicine [10,14,28]. Familial hypercholesterolemia is underdiagnosed, and there is limited uptake of cascade testing in the United States [3,29,30]. FH Family Share was designed to facilitate communication between familial hypercholesterolemia probands and their relatives, and to increase the uptake of cascade testing. It was iteratively refined through usability testing with genetic counselors and patients with familial hypercholesterolemia. The target sample size for participant recruitment was achieved in both phases of the pilot testing program, and based on study team consensus, additional participants were not recruited as feedback saturation was reached and majority of the usability issues had been identified.

Current guidelines [31,32] for familial hypercholesterolemia screening recommend the identification of a proband and cascade testing of family members, starting with first-degree relatives. Cascade testing for familial hypercholesterolemia has been identified as the most cost-effective strategy for identifying new familial hypercholesterolemia cases [33,34]. In traditional cascade testing, a proband sends a templated letter, using postal mail, to inform relatives of their diagnosis and to encourage them to get tested; however, there is no way to determine whether relatives receive the letter and seek testing for familial hypercholesterolemia. This approach to cascade testing has low uptake in the United States, in part due to the inability of health care providers to directly contact family members and the burden placed on probands to undertake this task, and in part due to the lack of an established national program with cascade testing that is centralized, coordinated, and aligned with local and regional needs and resources [35,36]. Although countries with nationalized health care systems have been able to implement centralized cascade testing programs, the United States is limited by several barriers. Well-designed usable digital tools can be used to bridge the gap in communication and increase uptake of cascade testing.

Usability testing is an integral part of the field of human–computer interaction and is used to evaluate interactive health apps to ensure effective design and development strategies, with a particular focus on the concept of user-centered design [37-41]. Genetic counselors and patients with familial hypercholesterolemia are the intended end users of the web-based tool, and feedback from these groups informed iterative refinements to FH Family Share after each phase of testing. Our extensive usability testing with genetic counselors revealed FH Family Share to be a resource worth returning to, with easy to find and easy to understand content that would likely improve patient follow-up care. Genetic counselors indicated that the tool was something they could share on their computer screens with patients during pre- and post-genetic testing counseling sessions. Genetic counselors also highlighted that the template email would assist patients in sharing their familial hypercholesterolemia diagnosis with relatives but that it needed to include sections in which either they (the genetic counselors) or the patients could insert gene and variant names, as this would be useful to family members when scheduling testing. Orlando et al [42,43] employed a similar process to develop a family health history and decision support tool, in which usability testing conducted with 10 genetic counselors resulted in a number of adaptations to the final tool, such as changes to interface design, navigation, and content, prior to its deployment.

Usability feedback from patients with familial hypercholesterolemia revealed that the majority of patients found content easy to understand, agreed that the tool could help them share their familial hypercholesterolemia diagnosis with family members, and was a resource worth returning to. A novel insight gained from patient usability testing was the importance of understanding the motivation for patients seeking genetic testing for familial hypercholesterolemia. Patients revealed that concern for their children, grandchildren, and family planning were the primary motivators. They highlighted that FH Family Share should contain an education module focused on considerations for children and family planning. This feedback led to the addition of a new topic module. Similar feedback was obtained in a study [44] conducted to identify motivators and barriers to cascade testing in families with familial hypercholesterolemia. Study participants indicated that they informed relatives of their risk of familial hypercholesterolemia to protect them from heart disease and allow them to make appropriate lifestyle changes [44].

FH Family Share will be implemented at all Mayo Clinic sites and the Mayo Clinic Health System and will also be available for public access on the internet. Once deployed, the tool’s content and resources will be updated as new knowledge emerges. The impact and metrics of FH Family Share will be assessed using a pilot implementation study. The findings described herein could be used to create similar digital apps for other genomic disorders.

### Limitations

Usability testing sessions were limited to genetic counselors and patients with familial hypercholesterolemia—input from family members of patients with familial hypercholesterolemia would be useful in ensuring effective implementation of the tool in nonclinical settings. Additionally, FH Family Share was not integrated with the electronic health record to enable ease of access in remote or underserved communities which may have different electronic health record systems in place. In some instances, feedback obtained from genetic counselors and patients with familial hypercholesterolemia varied on the same topic, for instance, most patients indicated the need for more illustrations on FH Family Share to increase engagement and interest, while genetic counselors suggested limiting the use of illustrations unless they served a specific purpose; feedback from both groups was harmonized by adding curated illustrations to increase patient engagement. Due to the COVID-19 pandemic, usability testing sessions conducted with patients were virtual and nonverbal cues could not be observed; however, screen sharing along with open-ended questions allowed us to obtain sufficient feedback which was often consistent across participants. The sample sizes for genetic counselors and patients with familial hypercholesterolemia were modest; however, the numbers were in line with those of previous usability studies [22-25] and allowed for thematic saturation to be reached.

### Conclusions

FH Family Share can be used by probands to communicate their familial hypercholesterolemia diagnosis with family members, encouraging them to seek testing for familial hypercholesterolemia. Such a tool has the potential to increase uptake of cascade testing thereby allowing for earlier detection and treatment of familial hypercholesterolemia.

## Appendix

Multimedia Appendix 1 Task scenarios used for the cognitive walkthrough conducted by usability experts.

Multimedia Appendix 2 Design and development specifications of FH Family Share.

Multimedia Appendix 3 Case scenarios.

Multimedia Appendix 4 Satisfaction survey (genetic counselors).

Multimedia Appendix 5 Survey (patients).

Multimedia Appendix 6 Themes and representative quotations.

Multimedia Appendix 7 Demographic characteristics (genetic counselors).

Multimedia Appendix 8 Demographic characteristics (patients).
