# Supplementary material for: Web-Based Tool (FH Family Share) to Increase Uptake of Cascade Testing for Familial Hypercholesterolemia: Development and Evaluation
Source: JMIR Hum Factors. 2022 Feb 15;9(1):e32568. doi: 10.2196/32568 (PMC8889478; doi:10.2196/32568)
Supplement: Multimedia Appendix 1 [file humanfactors_v9i1e32568_app1.docx]

# **Multimedia Appendix 1**

**Table:** Task scenarios used for the cognitive walkthrough conducted by usability experts.

| **Task** | **Scenario** |
| --- | --- |
| 1 | You would like to learn more about familial hypercholesterolemia (FH), please log in to the website. |
| 2 | You have successfully logged in and now want to learn about the symptoms of familial hypercholesterolemia (FH). Please locate this information. |
| 3 | You are interested in finding out about ongoing research involving familial hypercholesterolemia (FH), where would you go to find this? |
| 4 | You have recently been diagnosed with familial hypercholesterolemia (FH) and would like to share some information on your diagnosis with your family members. How would you do this? |
| 5 | You are interested in inviting a family member to use the website. Please enter the following email address and send an email invitation. |
| 6 | You have some questions regarding familial hypercholesterolemia (FH) and want to look for your answers. Where would you find these? |
| 7 | You are interested in creating a family tree to keep track of relatives that may be at risk for familial hypercholesterolemia (FH). Please use the family tree tool to create a family tree that includes two parents and one sibling. |
| 8 | You are interested in taking a quiz to see if you are at risk for familial hypercholesterolemia (FH), how would you do this? |
| 9 | You would like to look up additional resources such as the FH Foundation website. Please locate this. |
| 10 | You are interested in reading publications that relate to familial hypercholesterolemia (FH). Please locate them. |
| 11 | You would like to participate in the Mayo Clinic FH research program. Please send an email to the study coordinator informing them of your participation. |
