# Supplementary material for: Web-Based Tool (FH Family Share) to Increase Uptake of Cascade Testing for Familial Hypercholesterolemia: Development and Evaluation
Source: JMIR Hum Factors. 2022 Feb 15;9(1):e32568. doi: 10.2196/32568 (PMC8889478; doi:10.2196/32568)
Supplement: Multimedia Appendix 2 [file humanfactors_v9i1e32568_app2.docx]

# **Multimedia Appendix 2**

### **Design and Development Specifications of FH Family Share**

Findings from the cognitive walkthrough led to the web-tool prototype being rebuilt by an institutional information technology (IT) team to align it with Mayo Clinic standards. FH Family Share was rebuilt using Angular 6 and MVC (5.2.6) to write the front end of the website, while the backend was built using C# .Net (4.7) and WebApi (5.2.6). FH Family Share was designed to have a contemporary user interface with a responsive design that displays static content and can be accessed via smartphone, tablet, laptop, or desktop. It has been optimized for search engines with the addition of specific keywords and descriptions on different web pages and is compatible with several web browsers including Chrome, Safari, Firefox and Internet Explorer 9+. The medical content for FH Family Share was generated by an expert physician in the field of FH and preventive cardiology and will be updated as research and guidelines evolve.

The About Me pedigree tool available in FH Family Share was developed independently of the web-tool by Mayo Clinic. Users interact with About Me inside of an I-Frame in FH Family Share. The About Me front end was developed using Angular JS (1.5.5) with WS-Federation for user authentication and the backend was developed using C# .Net (4.5.2) and WebApi (5.2.3) and a SQL Server Relational Database. The About Me application sits behind the Mayo Clinic patient portal which ensures that all protected health information is secure. The pedigree tool is also linked to other patient services, ensuring a smooth user experience.
