# Supplementary material for: Web-Based Tool (FH Family Share) to Increase Uptake of Cascade Testing for Familial Hypercholesterolemia: Development and Evaluation
Source: JMIR Hum Factors. 2022 Feb 15;9(1):e32568. doi: 10.2196/32568 (PMC8889478; doi:10.2196/32568)
Supplement: Multimedia Appendix 3 [file humanfactors_v9i1e32568_app3.docx]

# **Multimedia Appendix 3**

**Table 1:** Two case scenarios were presented to each genetic counselor and the Think Aloud usability technique was applied to navigate the prototype.

| **Case Scenarios** |
| --- |
| *Case 1:* Dan, a 42-year-old male, is on-site and came in for an evaluation of elevated cholesterol levels. He was found to have a low-density lipoprotein receptor (*LDLR*) pathogenic variant. It is now time to discuss the results with Dan and the impact on family members who may need to be tested. How might you introduce and describe this website to Dan? Are there notes or comments you would share with Dan as to how to get the most out of the website? |
| *Case 2:* Dan’s 33-year-old sister Kay is at home and receives an email from Dan; the email informs her of Dan’s familial hypercholesterolemia (FH) diagnosis and recommends she get tested for FH as it runs in families. How do you feel Kay could make the best use of the FH Family Share website? |
