# Supplementary material for: Web-Based Tool (FH Family Share) to Increase Uptake of Cascade Testing for Familial Hypercholesterolemia: Development and Evaluation
Source: JMIR Hum Factors. 2022 Feb 15;9(1):e32568. doi: 10.2196/32568 (PMC8889478; doi:10.2196/32568)
Supplement: Multimedia Appendix 4 [file humanfactors_v9i1e32568_app4.docx]

# **Multimedia Appendix 4**

**Table:** In both phase I and II of the pilot testing program, a satisfaction survey was completed by genetic counselors at the end of the usability testing sessions.

| **Survey Questions** |
| --- |
| **Phase I Survey Questions:**   1. Overall, the information that you were asked to assess within the FH Family Share website was:  - Very easy to find - Somewhat easy to find - Neither easy nor difficult to find - Somewhat difficult to find - Very difficult to find  1. Overall, the information that you found within the FH Family Share website was:  - Very easy to understand - Somewhat easy to understand - Neither easy nor difficult to understand - Somewhat difficult to understand - Very difficult to understand  1. Is the FH Family Share website a resource worth returning to?  - Yes - No  1. What did you like most about the FH Family Share website? (*free text*) 2. What did you like least about the FH Family Share website? (*free text*) 3. What additional information or functionality would you like to see on the FH Family Share website? (*free text*) 4. What more could we do to improve the FH Family Share website? (*free text*) |
| **Additional Questions Added in Phase II:**   1. The FH Family Share website will ease my workflow in a patient encounter:  - Completely Agree - Agree - Neither Agree nor Disagree - Disagree - Completely Disagree  1. The FH Family Share website is likely to improve follow up patient care:  - Completely Agree - Agree - Neither Agree nor Disagree - Disagree - Completely Disagree  1. As a provider I feel the patient is most likely to (circle one option):  - Use the Learn modules as a knowledge resource - Build a family tree using AboutMe - Calculate risk of heart attack - Send a letter to family members - Use website information to discuss FH with family members  1. Do you find the FH Family Share website Figures/Images/Diagrams useful?  - Yes - No - Other: |
