# Supplementary material for: Web-Based Tool (FH Family Share) to Increase Uptake of Cascade Testing for Familial Hypercholesterolemia: Development and Evaluation
Source: JMIR Hum Factors. 2022 Feb 15;9(1):e32568. doi: 10.2196/32568 (PMC8889478; doi:10.2196/32568)
Supplement: Multimedia Appendix 5 [file humanfactors_v9i1e32568_app5.docx]

# **Multimedia Appendix 5**

**Table:** An 11-item survey was completed by patients at the end of the usability testing sessions.

| **Survey Questions** |
| --- |
| **Phase I Survey Questions:**   1. Overall, the information that you were asked to assess within the FH Family Share website was:  - Very easy to find - Somewhat easy to find - Neither easy nor difficult to find - Somewhat difficult to find - Very difficult to find  1. Overall, the information that you found within the FH Family Share website was:  - Very easy to understand - Somewhat easy to understand - Neither easy nor difficult to understand - Somewhat difficult to understand - Very difficult to understand  1. The FH Family Share website will make it easier for me to share information with my family:  - Completely Agree - Agree - Neither Agree nor Disagree - Disagree - Completely Disagree  1. The FH Family Share website is likely to improve my care:  - Completely Agree - Agree - Neither Agree nor Disagree - Disagree - Completely Disagree  1. As a patient I feel I am most likely to (circle one option):  - Use the Learn modules as a knowledge resource - Build a family tree using AboutMe - Calculate risk of heart attack - Send a letter to family members  1. Do you find the FH Family Share website Figures/Images/Diagrams useful?  - Yes - No - Other (*free text*)  1. Is the FH Family Share website a resource worth returning to?  - Yes - No  1. What did you like most about the FH Family Share website? (*free text*) 2. What did you like least about the FH Family Share website? (*free text*) 3. What additional information or functionality would you like to see on the FH Family Share website? (*free text*) 4. What more could we do to improve the FH Family Share website? (*free text*) |
