# Supplementary material for: Web-Based Tool (FH Family Share) to Increase Uptake of Cascade Testing for Familial Hypercholesterolemia: Development and Evaluation
Source: JMIR Hum Factors. 2022 Feb 15;9(1):e32568. doi: 10.2196/32568 (PMC8889478; doi:10.2196/32568)
Supplement: Multimedia Appendix 6 [file humanfactors_v9i1e32568_app6.docx]

# **Multimedia Appendix 6**

**Table:** Themes and representative quotes identified from the cognitive walkthrough conducted by usability experts on the FH Family Share prototype.

| **Theme** | **Representative Quotes** |
| --- | --- |
| Design | “Find graphics that will help the user understand the concepts and use the graphics wisely.”  “Check all contrast ratios…”  “The first call to action box is filled with all caps which makes it hard to read.” |
| Format | “Is it necessary to have all caps in the top navigation elements?”  “The contact page seems to want to limit what I can ask questions about...there could be a form for ‘Contact Us.’” |
| Navigation | “Many of the reference links to materials (e.g. FAQ) send me to new tabs and large content.”  “After working on the site, the user could have 15 tabs open which reduces efficient navigation.” |
| Terminology | “Actions are not intuitive. If I select a letter to family members, I get a new tab and pdf.”  “Who is the audience for this information, they look like journal articles that many might not want to wade through.” |
| Instructions | “No indication that I am logged in.”  “Not sure what the family tree does for me except provide information that I construct.” |
| Learnability | “The evaluation resulted in several findings, some of which might fluster the user.” |
