# Supplementary material for: Web-Based Tool (FH Family Share) to Increase Uptake of Cascade Testing for Familial Hypercholesterolemia: Development and Evaluation
Source: JMIR Hum Factors. 2022 Feb 15;9(1):e32568. doi: 10.2196/32568 (PMC8889478; doi:10.2196/32568)
Supplement: Multimedia Appendix 7 [file humanfactors_v9i1e32568_app7.docx]

# **Multimedia Appendix 7**

**Table:** Demographic characteristics of genetic counselors who participated in the pilot testing program.

| **Genetic Counselors** | **Phase I**  **n (%)** | **Phase II**  **n (%)** |
| --- | --- | --- |
| Sex (females) | 9 (100) | 7 (100) |
| Age  20-29  30-39  ≥ 40 | 5 (56.0)  2 (22.0)  2 (22.0) | 5 (71.4)  1 (14.3)  1 (14,3) |
| Years of experience  <5  5-10  > 10 | 5 (56.0)  2 (22.0)  2 (22.0) | 5 (71.4)  1 (14.3)  1 (14.3) |
| Area of expertise  Cancer genetics  Cardiology  Education and prenatal counselling  Personalized medicine  Predictive genomic testing | 4 (44.4)  2 (22.2)  1 (11.1)  1 (11.1)  1 (11.1) | 4 (57.1)  2 (28.6)  1 (14.3)  0 (0.0)  0 (0.0) |
| Department  Clinical genomics  Center for individualized medicine | 6 (67.0)  3 (33.0) | 6 (86.0)  1 (14.0) |
