# Supplementary material for: Web-Based Tool (FH Family Share) to Increase Uptake of Cascade Testing for Familial Hypercholesterolemia: Development and Evaluation
Source: JMIR Hum Factors. 2022 Feb 15;9(1):e32568. doi: 10.2196/32568 (PMC8889478; doi:10.2196/32568)
Supplement: Multimedia Appendix 8 [file humanfactors_v9i1e32568_app8.docx]

# **Multimedia Appendix 8**

**Table:** Demographic characteristics of FH patients (n=9) who participated in the FH Family Share usability testing sessions.

| **FH Patients** | **n (%)** |
| --- | --- |
| Sex  Females  Males | 8 (88.9)  1 (11.1) |
| Age  30-39  40-49  ≥ 50 | 3 (33.3)  3 (33.3)  3 (33.3) |
| Race/Ethnicity  Non-Hispanic White | 9 (100) |
| Highest Level of Education  High school  Undergraduate  Graduate | 1 (11.1)  7 (77.8)  1 (11.1) |
| Occupation Related to Field of Medicine  Physician Assistant  Registered Nurse | 1 (11.1)  1 (11.1) |
| FH Variant Identified  *LDLR*  *APOB*  *PCSK9* | 4 (44.4)  5 (55.6)  0 (0.0) |
